# Effects of Nutritional Factors on Fat Content, Fatty Acid Composition, and Sensorial Properties of Meat and Milk from Domesticated Ruminants: An Overview

**DOI:** 10.3390/ani14060840

**Published:** 2024-03-08

**Authors:** Eric N. Ponnampalam, Hasitha Priyashantha, Janak K. Vidanarachchi, Ali Kiani, Benjamin W. B. Holman

**Affiliations:** 1 School of Agriculture, Food and Ecosystems Sciences, The University of Melbourne, Parkville, VIC 3010, Australia; 2 Formerly Agriculture Victoria Research, Department of Jobs, Precincts and Regions, Bundoora, VIC 3083, Australia; 3 Department of Molecular Sciences, Swedish University of Agricultural Science, SE-750 07 Uppsala, Sweden; 4 Department of Animal Science, Faculty of Agriculture, University of Peradeniya, Peradeniya 20400, Sri Lanka; janakvid@pdn.ac.lk; 5 Animal Science Department, Faculty of Agriculture and Natural Resources, Lorestan University, Khorramabad 68151-44316, Iran; kiani.a@lu.ac.ir; 6 Wagga Wagga Agricultural Institute, NSW Department of Primary Industries, Wagga Wagga, NSW 2650, Australia

**Keywords:** sheep, goat, buffalo, camelid, dairy cow, beef, cheese, feeding system, nutritional value, fatty acid profile

## Abstract

**Simple Summary:**

Nutritional interventions in ruminant livestock management is an essential step to achieving high quality meat and milk products for diversified and competitive global markets. The alterations of fat content, fatty acid profile, and the associated sensory properties of meat and milk have attracted much attention; they are accomplished with the managing of feeding systems and nutrition of ruminant diets. The literature has detailed various feed types and ingredients that facilitate the sustainable use of abundant, novel by-products, secondary products, non-conventional feedstuffs, or minimally processed biological materials within ruminant farming systems. Relevant to major feed ingredients is the knowledge of their macro- and micronutrients, as well as their bioactivity and functionality in meat and milk products. This review examines these and provides an overview of various concentrate feeds and forages that are fed to ruminants, and how they relate to the fat content and fatty acid profile of their meat and milk products. These insights will be valuable to those seeking to understand and adopt nutritional measures for the enhancement of domesticate ruminant meat and milk products.

**Abstract:**

The meat and milk products from domesticated ruminants are important foods within a balanced diet, offering a rich source of energy, protein, fats, minerals, and vitamins. The sensorial properties of meat and milk are mainly linked to their fat content and fatty acid composition, which are influenced by the feeding background or nutrient composition of diets. While several studies have investigated the nutritional effects on the fat content and fatty acid profile of ruminant meat and milk, as well as their relationship with sensorial properties, a comprehensive overview of these effects is lacking. This paper summarises current literature and discusses changes to fatty acid composition (including ω-3 concentrations), fattiness, and associated quality traits of sheep, goat, beef cattle, alpaca, and llama meat that can be achieved by using different forages or feeds in a total mixed ration. Additionally, it presents the shelf life and nutritional value of meat, milk, and cheeses from the milk of dairy cattle, buffalo, goats, and sheep as influenced by a ruminant diet. Further advancement in these areas will promote the sustainability of ruminant production and its associated feeding systems in achieving premium quality animal-derived foods.

## 1. Introduction

Meat and milk are important sources of nutrients, from childhood to old age, for many people from around the world. It is well documented that quality meat and milk and their processed products promote consumer satisfaction and nutritional requirements while alleviating the development of metabolic syndromes and various disease risks [1]. The nutrient composition of meat and milk is influenced by the type of diets that the animals consume. For example, feeding concentrate diets (feedlot) containing high proportions of grains to sheep may increase carcass fatness, ω-6 fatty acid (FA) concentrations, and the ratio of ω-6/ω-3 in meat [2]. On the contrary, allowing dairy cattle to graze fresh pasture improves the concentration of beneficial fatty acids such as ω-3 FAs and conjugated linoleic acids (CLAs) in milk [3]. Another example is that diets rich in ω-3 FAs contribute to less milk wastage via their reduction of free radicals and the number of somatic cells in milk, reducing the occurrence of udder infections [4]. The latter effects are associated with the composition and nutrient availability of feeds that further influence ruminal biohydrogenation process, lipid hydrolysis, digestion, and the absorption of nutrients in the enterocyte of the host animal. Subsequently, it enhances the metabolic activities in the body, thereby determining the availability of energy and nutrients for the formulation of meat and milk products.

Ruminant animals (i.e., cattle, buffalo, goats, sheep, alpacas, and llamas) are evolutionary habituated species that can convert the abundantly available pastures, fodders, silage, hay, crop residues, brewery waste, and by-products of agriculture industries into valuable products, i.e., meat, milk, wool, offal, and leather. The nutritional requirements and dietary regulations of ruminant animals are important for optimal animal performance and product quality. Numerous studies have investigated the effect of feeding systems and feed composition on the yield, quality, and composition of meat and milk in ruminant animals [5,6]. This comprehensive review covers fat content, fatty acid composition, and sensory attributes (e.g., taste and flavour) of meat and milk from sheep, cattle, buffalo, goats, alpacas, and llama. These quality attributes of meat and milk are influenced by livestock management practices, nutritional background, animal genetics, and processing technologies. Among these factors, the nutritional background has been found to play a crucial role in altering the fat level, fatty acid composition, and sensorial attributes of meat and milk from ruminants. Therefore, the scope of this review is limited to the effect of nutritional factors.

The variation in the fat content, fatty acid composition, and flavour of meat and milk is mainly influenced by the macronutrient, such as protein, fat, and carbohydrate (energy), concentrations of the diet and their digestion and absorption process. The minerals, vitamins, and bioactive components from the diets further help to maintain the cellular systems through protective mechanisms and defence actions (e.g., antioxidant action of the body and its defence against free radical formation) and, therefore, maintaining the quality characteristics of lipids and fats in meat and milk.

Ruminants have ecologically evolved to live outdoors (extensive farming systems) and harvest forage materials from rangelands for their production and survival. Rearing ruminants under pasture or forage feeding is economical, but it does not always produce superior weight or better animals with greater fatness when compared with concentrate (e.g., total mixed ration; TMR) or feedlot finishing. However, with advances in agricultural industries and technologies in the modern era, ruminant animals are increasingly housed under intensive or semi-intensive systems that better suit faster growth rates, environmental benefits, and animal welfare practices [7]. In addition to fresh forages, silage, and hay, ruminants are fed with field-crop residues, cereal grains, protein meals, and by-products from agrifood industries when reared under intensive or semi-intensive production systems. Such a practice is called lot feeding, feedlotting, concentrate feeding, or supplementary feeding. These systems are generally more expensive than extensive production (grazing) and contribute to more fat deposition in the body, carcass, or primal cuts in meat-producing animals and more milk yield or less milk solid fat content in dairy animals. This, in turn, may alter the fat composition and sensory properties of ruminant animal meat and milk products.

The FA profiles of meat and milk from ruminants have proven to efficiently discriminate between diets fed to finishing lambs, goats, and beef cattle as well as lactating dairy ewes, does, and cows. Furthermore, meat and milk lipids or FAs are directly linked to their organoleptic (e.g., aroma, taste, and mouthfeel) properties and nutritional aspects that fulfill consumer desires and willingness to purchase [8]. In meat, intramuscular fat (IMF) is reported to have higher concentrations of polyunsaturated fatty acids (PUFAs) compared to subcutaneous and visceral fat deposits [9], although efforts to increase IMF concentrations in lamb have resulted in a relative increase in monounsaturated and saturated fat contents (e.g., C18:1, C14:0) and branched-chain FAs [10]. The relationship between IMF and specific FAs is the likely pathway by which IMF is associated with meat sensory properties [11,12]. In addition, the changes in the FA composition of milk through feeding strategies can also alter its flavour and consumer appeal.

It has been well documented that ruminant farm animals grown under extensive systems are smaller in size, lighter in weight, and have less body fat with proportionately greater levels of SFAs, ω-3 FAs, rumenic acid (RA, C18:2 cis-9,trans-11 known as CLA) and trans-vaccenic acid (TVA, C18:1 trans-11) in body tissues, whereas the animals reared in intensive or semi-intensive systems and fed with grain-based concentrate diets are larger in size, have a heavier weight, and greater body fat with proportionately greater levels of oleic acid, ω-6 FAs and ω-6/ω-3 in body tissues [13,14]. Furthermore, ruminant animals fed with supplements or by-products of the biofuel and oil industries (e.g., corn, sunflower, safflower, cotton, soybean, rape, canola, camelina, flax) have been reported to have altered levels of fatness and fatty acid composition (ω-3 FAs, ω-6 FAs, CLAs, and oleic acid) in meat [15,16,17,18,19,20] and milk [5,21,22,23].

**Figure 1:**
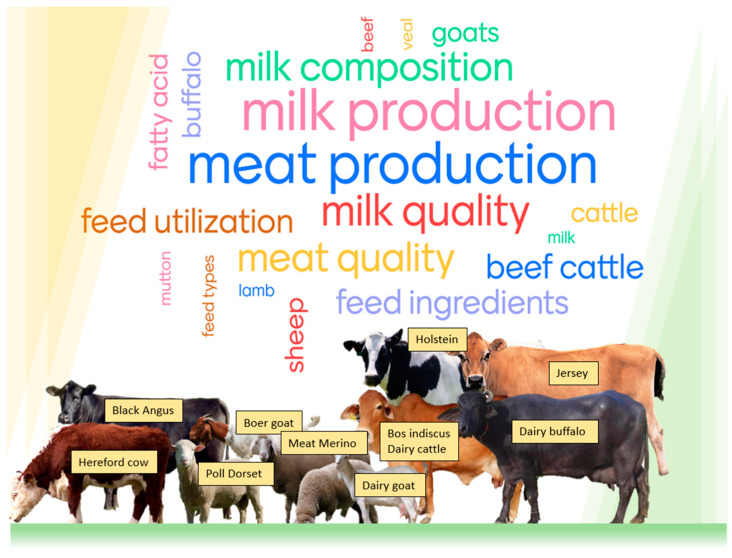
A word cloud of the common ruminant products and influential nutrition factors for the production of sheep, goats, cattle, buffalo, and alpaca.

There are many reasons for the variation in fat level, fat composition, and the sensorial attributes of meat and milk produced from ruminants grown under different production systems. Some examples are the type of feed, lipid/fat level in the feed, crude protein concentration and type of protein in the feed, fibrous nature (bulk) of feed, feed selection behaviour of animal, the energy expenditure of animals to walk and harvest feed materials, etc. It is known that, based on the structural carbohydrates (fibre), fat and protein concentrations of feeds (macronutrients), the formation of short-chain fatty acids (acetate, propionate, and butyrate), and production of methane gas in the rumen vary. This, in turn, can determine ruminant productivity, the fat content, and taste of meat and milk. Furthermore, in the last decade, there has been increased interest in altering meat and milk nutritional components to enhance their content of essential FAs and antioxidants and promote better human health and well-being [24]. With this intention, strategic management of ruminant animal diets has gained considerable attention and modification. Novel knowledge is constantly emerging by investigating the possibilities of including various types and levels of feed ingredients that are suitable to the agroecological regions and production systems and, thus, creating topical interest in evaluating these cases for various species of ruminant animals that produce milk and meat. This review aimed to provide an overview of various diets and feeding strategies (nutritional background) contributing to the level, composition, and taste (flavour and aroma) of fat in meat and milk from the ruminant animals shown in Figure 1.

## 2. Nutritional Effects on the Meat of Sheep, Goats, and Cattle

Sheep meat is categorised as lamb, hogget, or mutton, based on its age when slaughtered [25]. Likewise, goat meat is categorised by age at slaughter, as either mutton or chevon. Lamb and mutton remain niche components of consumer diets in many societies, restaurants, and markets compared to meat from other farm animals. This is because of their particular flavour profile and texture that are associated with the fat level and fatty acid composition. Sheep and goats have evolved to consume a wide range of feeds such as grasses, fodders, shrubs, and the vegetative parts of small and large trees and their foliage. This differs from larger ruminants that mainly consume pasture, fodders, and preserved materials, such as haylage and silage or roughages, and hay from cereal crop production [26]. As a rule of thumb, sheep meat is more popular in developed and developing countries, while goat meat and milk are more popular in developing countries. Compared to cattle and pigs, the products from sheep and goats are not subjected to cultural/religious constraints, are easy to handle in smallholder farming systems, demand fewer resources associated with environmental constraints to grow, and have a high nutrient value of meat and milk. Due to these reasons, although the share of global protein consumption is lower than beef, the demand for sheep meat and goat meat around the world is expected to grow in the future [27]. The major sheep meat-producing nations include China, New Zealand, Australia, the European Union, and the United Kingdom. The major goat meat-producing nations include China, India, Pakistan, Nigeria, and Bangladesh.

**Figure 2:**
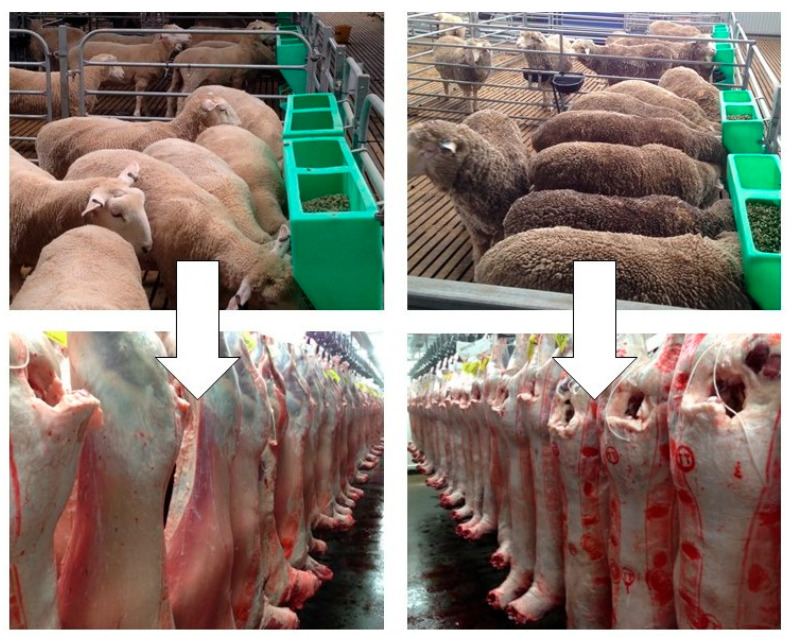
Appearance of crossbred lambs (**left**) and Merino yearlings (**right**) and their carcasses when fed the same feedlot finisher pellets for a 9-week period [30]. Agriculture Victoria are acknowledged, being where this feeding study was conducted.

Sheep and goats may be termed small ruminants. Generally, rumen biohydrogenation, digestion, and nutrient absorption processes in small ruminants are similar to that of larger ruminants, when fed with diets of a similar nutritional background. As a result, sheep and goats have been used as a model for large ruminants in many research aspects (e.g., fat deposition or product quality). However, we note that the efficiency of nutrient partition and fat–meat tissue deposition may vary between breeds due to differences in feed intake, particle selection, and chewing process associated with animal body size, body structure, and production purpose [28,29]. A recent study (Figure 2) shows the differences in animal body structure, carcass conformation and fat–muscle deposition of 6–7-month-old, crossbred lambs used for meat purposes and 16–17-month-old yearling sheep used for dual purposes (wool–meat), respectively. In this study, both lambs and yearling sheep were offered the same feedlot pellets consisting of metabolizable energy, crude protein, lignin, and NDF contents at 10.8 MJ, 150 g, 43 g, and 340 g/kg dry matter, respectively. Feed was offered at ad libitum level over a 9-week feeding period followed by a slaughter. The fat and lean content of carcasses for crossbred lambs and yearling sheep as assessed by DEXA were 34 vs. 37 and 64 vs. 62 percentages, respectively (see Figure 2).

### 2.1. Sheep and Lamb Meat

In production systems around the world, where fresh pasture is insufficient to finish sheep for meat production, it is common that farmers use concentrate diets (feedlot), grains, or protein meals containing high energy and protein as supplements to attain the desired weight at slaughter [31]. These strategies result in animals delivering larger carcasses at a younger age when compared with pasture feeding solely.

It is known that, based on the demographic regions people live in, different populations prefer meat from animals slaughtered at different ages or different live weights [32,33,34,35]. For example, North Americans and Australians prefer meat cuts from carcasses weighing more than 22 kg, slaughtered at 6–12 months of age; European consumers (Spain, France, and Italy) prefer meat cuts from light lambs weighing less than 18 kg carcass weight, slaughtered at less than 4–6 months of age; and Middle Eastern and Asian Pacific region consumers prefer sheep meat from older animals, slaughtered at more than 12–18 months of age. The reason is that the consumers of some countries prefer meat with minimal fat and more muscle (light lamb), some countries prefer tender and juicier meat cuts with considerable fatness/marbling (trade to heavy lamb), and others prefer meat from mature animals, especially for the chewy meat with a distinctive mutton flavour (yearling sheep).

**Figure 3:**
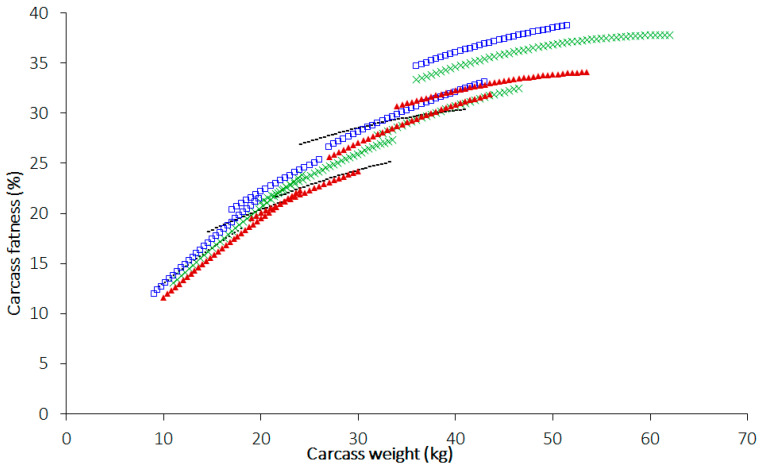
The relationships between carcass fat content (%) and carcass weight (kg) for Merinos (**----**), Border Leicester Merino first cross (BL × M = □□□□), Poll Dorset Merino first cross (PD × M = ∆∆∆∆), and Poll Dorset × Border Leicester Merino first cross (PD × BLM = ××××). For a genotype, there are four disconnected lines with the same drawing symbol representing, from left to right, slaughtered at 4, 8, 14, and 22 month of age.

Carcass fatness refers to intramuscular (marbling), intermuscular, and subcutaneous fats and is different between breeds of sheep, as well as influenced by the nutrient (protein, fat, and energy) concentrations of the diet and the length of feeding. If one would like to understand the fat accretion in the body among different breeds at different ages, the animals should be grown under the same nutritional plane for comparisons of carcass fatness. A study conducted in New Zealand in the 1990s showed the differences in carcass fatness and muscle composition of sheep breeds covering meat-type, wool-type, and dual-purpose-type sheep [36]. A detailed study conducted in Australia [28] reports the carcass fat content compared at sucker (4 months), lamb (8 months), yearling (14 months), and mutton (22 months) ages, covering all traditional breeds used for meat, wool, and meat–wool purposes (see Table 1 for details). The latter study also examined the carcass fatness among all breeds on a common carcass weight basis (see Figure 3 for details), i.e., at 10, 20, 30, and 40 kg carcass weight [37]. The study revealed that carcass fatness can vary between breeds at a particular weight, which is associated with the metabolic requirement, energy partition as adipose/muscle tissues, and productive status of the animal. For example, second cross (Poll Dorset × Border Leicester) × Merino lambs at 4 months were equal to pure Merino × Merino cross at 8 months of age. Similarly, first cross Border Leicester × Merino lambs at 8 months of age had similar carcass fatness to first cross Merino × Merino yearlings at 14 months of age [37]. Data presented in Figure 3 and Table 1 reveal that carcass fatness contributing to intramuscular, intermuscular, and subcutaneous fat vary substantially among breeds and ages of animals, and this can impact the fatty acid composition of meat resulting in different flavours, texture, and juiciness of meat.

Carcass characteristics and the shelf life of meat have a significant role in delivering quality and healthy food to consumers of interest. Considering the increase in meat consumption among the population in the last two decades, the important role of meat in the human diet, and the concerns that have arisen regarding the consumption of fat from meat and its impact on human health, several studies have focused on ways of improving fat content and FA composition of meat in farm animals [16,19,38,39,40]. In this context, when sheep are fed with pasture and forages (grass silage and haylage), their meat tends to have a greater concentration of ω-3 FAs and CLAs and a lower ratio of ω-6/ω-3 to a level that is nutritionally favourable. In addition, meat also tends to have a greater concentration of SFAs, which is undesirable from the perspective of human health [41,42,43]. In contrast, when sheep, goats, or cattle are fed with grain supplements or concentrate diets high in grains, their meat is greater in fatness with increased levels of C16 and C18 MUFAs, and C18 and C20 ω-6 FAs [38,44,45,46].

#### 2.1.1. Sensory Properties and Types of Flavours

Sensory studies conducted in lamb and mutton from sheep and chevon from goats report many flavour types and the main ones are lamby, muttony, pastoral, livery, metallic, grassy, bloody, cardboard, and greasy based on the fat level and development of volatile compounds during cooking [39,47,48,49,50,51]. The acceptance level of taste (flavours) varies with populations from different demographic regions, country of origin, ethnic groups, and religious backgrounds. For example, consumers from Middle Eastern and Asian countries prefer the flavour and aroma of meat from older (aged more than 18–24 months) sheep and goats due to customs, availability and affordability [27]. Consumers and markets from developed countries such as the United Kingdom, United States, and Australia prefer meat from young sheep called lamb due to its high-quality cuts offering juicy, tender, and lamby flavour [52]. Other than animal age, the lamby flavour is associated with several factors such as diet, gender, breed, and duration of feeding [47]. Some flavours are desirable and acceptable by consumers, while others are categorised as off-flavours or taints. The taste of meat is also dependent on the cooking method to some extent and this has been discussed in detail by Schwartz et al. [53] and Yancey et al. [54].

#### 2.1.2. Polyunsaturated Fatty Acids and Flavour Properties

In general, consumer satisfaction with meat flavour is negatively correlated with PUFAs, mostly associated with the oxidation of longer-chain ω-3 FAs and ω-6 FAs [55,56,57]. Nevertheless, Francisco et al. [58] identified a positive correlation between PUFA concentration in meat and sensory attributes. The taste of meat is altered due to the increased concentrations of ω-6 FAs and ω-3 FAs and their proportions in meat are believed to contribute to the physicochemical properties of lipids/fats or propagation of lipid oxidation from these FAs during storage and cooking process, yielding primary and secondary oxidative substances [59]. The literature has reported an increase in linoleic acid (C18:2n-6) in the muscles of animals when fed concentrate or grain-based diets containing cereal grains (oat, barley, and wheat), corn, or cottonseed [60,61,62]. A part of the linoleic acid is deposited as phospholipids and further enhances the production of longer-chain C20:3n-6, C20:4n-6, and C22:4n-6 through desaturation and elongation processes. Pasture-based diets such as grass or silage feeding produce meat with elevated levels of linolenic acid (C18:3n-3) mostly deposited as phospholipids. Linolenic acid further enhances the production of longer-chain ω-3 FAs of C18:4n-3, C20:5n-3, C22:5n-3, and C22:6n-3 in muscle tissues through the desaturation and elongation process [63,64]. Specialised feeds and mixed rations that contain oilseeds and oilseed meals, such as from flax, canola, and camelina, can increase linolenic acid and longer-chain ω-3 FA concentrations of the meat [14,65]. Furthermore, feeding marine-based products such as seaweeds, algae, fishmeal, and fish oil as supplements in sheep diets mainly increase concentrations of longer-chain ω-3 FAs of C20:5n-3, C22:5n-3, and C22:6n-3 in muscle tissues [16,62,66,67,68]. All these factors show how the changes caused by different basal diets and supplement combinations can develop different flavours, aromas, or taints reported in Section 2.1.1, due to cooking or other oxidation processes associated with storage conditions and the storage length of meat.

Lipid oxidation is a continuous chain reaction that occurs when unsaturated fatty acids react with oxygen and produce lipid hydroxides. These are unstable and will decompose into secondary oxidative products, such as LH, L, LOO, LOOH, and LO, that may, in turn, generate free radicals and infer malodourous and rancid flavour properties to meat [14].

The type of concentrate feed can affect the flavour of lamb meat by modifying its fatty acid profile. Nute et al. [69], for example, evaluated the flavour of meat from lambs fed concentrate diets supplemented with flax oil, fish oil, protected lipid supplement (PLS), fish oil with marine algae, or PLS with marine algae. The lambs fed with flax oil had the highest concentration of linolenic acid in their phospholipids and gave the highest ratings for meat flavour and overall liking, and a lower rating for abnormal lamb flavour, similar to the findings from other studies [55,70]. Diets supplemented with fish oil and PLS with marine algae produced meat with higher concentrations of ω-3 FAs (EPA and DHA) but increased levels of fishy and abnormal flavours while reducing the rating of lamb flavour. Sañudo et al. [71] showed that flavour intensity and flavour liking for lamb meat were strongly associated with linolenic acid concentrations while Fisher et al. [72] found that the concentration of linolenic acid in lamb meat increased with grass feeding when compared with concentrate feeding, whereby the score for lamb flavour intensity increased and that of abnormal flavour was reduced.

#### 2.1.3. Odd- and Branched-Chain Fatty Acids and Flavour Properties

Odd- (e.g., 15:0, 17:0) and branched-chain fatty acids (OBCFAs) are quantitatively minor FAs that can be found in meat and milk fat from ruminants [73]. Odd-chain fatty acids are synthesized from propionic acid and microbial de-novo lipogenesis [74]. The most abundant BCFAs in ruminant products are iso- and anteiso-mono-methyl BCFAs with a chain length from 14 to 17 carbon atoms. They are synthesised by the rumen microorganisms, specifically, bacteria and protozoa from dietary branched-chain amino acids (i.e., valine, leucine, and isoleucine). The final products of the BCFA biosynthetic pathway in the rumen are iso-14:0 and iso-16:0, derived from valine, iso-15:0 and iso-17:0 from leucine, and anteiso-15:0 and anteiso-17:0 from isoleucine [75]. BCFAs are key structural lipid constituents of the bacterial membrane. As bacteria cells are washed out from the rumen, their membrane undergoes digestion and the BCFAs are absorbed and subsequently incorporated into milk fat and other tissues [66]. The BCFA composition of milk from sheep, goats, and cows comprises approximately 1.8–3.1%, 1.2–2.4%, and 1.7–3.4% of total FAs, respectively [75]. The lipids of rumen bacteria differ between species; that said, cellulolytic bacteria usually contain more iso-form FAs, whereas amylolytic bacteria are mainly rich in linear odd-chain FAs, with a few strains displaying high contents of anteiso-form FAs [75,76].

Animal diets have been reported to influence the formation of OBCFAs [76]. The branched-chain fatty acids present in red meat such as 4-methyloctanoic acid (MOA), 4-ethyloctanoic acid (EOA), and 4-mythylnonanoic (MNA) are very small in concentration and hard to determine quantitatively; therefore, it is not well-known at what concentrations they can detrimentally affect the taste or flavour of meat from sheep, goats, or cattle. In this regard, recent studies were able to identify the dietary effects on MOA, EOA, and MNA concentrations in subcutaneous adipose tissue, since these branched-chain fatty acids are present in higher concentrations in subcutaneous adipose tissue [48,77] because it is easy to quantify. Further, it was observed that the subcutaneous fat layers or depots are mostly trimmed during carcass processing at the abattoir level, then during primal cut preparation at the retailer level, and finally during the cooking process. Frank, Watkins, Ball, Krishnamurthy, Piyasiri, Sewell, Ortuño, Stark, and Warner [48] reported that sheep fed perennial ryegrass, lucerne, and brassica forages had 220–393 μg MOA/g, 89–170 μg EOA/g, and 35.1–63.6 μg MNA/g concentrations in subcutaneous adipose tissue. In the meantime, different breeds of lambs fed intensively on concentrate diets had ranges at 56.9–103 μg MOA/g, 12.3–19.7 μg EOA/g, and 17.3–46.6 μg MNA/g concentrations in subcutaneous fat depots [77]. Young et al. [78] showed that lambs fed concentrate diets had greater levels of branched-chain fatty acids in subcutaneous adipose tissue than pasture-fed lambs due to the increased formation of propionic acid in the rumen of concentrate-fed lambs.

### 2.2. Chevon and Goat Meat

#### 2.2.1. Production System Effects on Chevon

**Figure 4:**
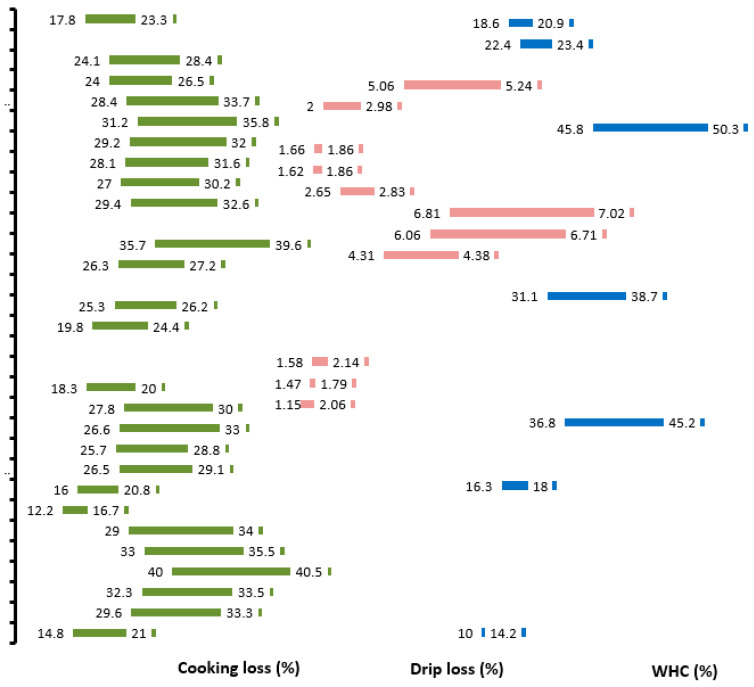
A summary of the ranges in cooking loss, drip loss, and water holding capacity (WHC) observed for goat meat (chevon), grown under different feeding systems. The values are reported in percentages (%) of weight change (loss) during the assessment of meat for cooking loss, drip loss, and water holding capacity.

It is evident from the literature presented above that the production system significantly influences fat content, FA composition, and taste of chevon [79,80,81,82,83]. In an intensive production system, goats have a heavier carcass weight, greater fat thickness, higher dressing percentage, and a lower ratio of unsaturated FAs to SFAs (UFA/SFA) compared with ones raised in semi-intensive production [79,80,81,82,84]. Kids raised on pasture are leaner and have a higher proportion of bone and lean-to-fat ratios compared to kids fed intensively [82,84]. However, Damascus male kids confined indoors and fed concentrate +200 g/day lentil hay (intensive) had similar slaughter and carcass traits as those allowed 8 h per day grazing and a 700 g/day concentrate in a semi-extensive, grazing system [83]. Meat from range-fed goats has a lighter colour [lower a*(redness), b*(yellowness)], greater off-flavour intensity and lower cooking loss compared to meat from concentrate-fed goats [84]. Water loss from muscle tissues pre-cooking and meat post-cooking are interconnected to the lean-to-fat ratio of the carcass and carcass cooling rate post-slaughter; both can be influenced by dietary regimes [85]. Figure 4 shows the variation in water-holding capacity, cooking loss, and drip loss reported in several studies of chevon from goats reared under intensive or extensive production systems, and supplemented with a concentrate. It is reasonable to state that feeding systems and strategies can influence fat and muscle composition in meat (and carcass) and these, in turn, are responsible for different ranges of water holding capacity, drip loss, and cooking loss in meat.

The inhalation of terpenes, such as limonene, associated with pasture forages, was found to result in their accumulation in the meat of goats, offering a pathway to the extension of shelf life and suppression of peroxidation and discolouration [86]. Regarding the FA composition, goats grazed in rangeland have higher UFA/SFA and lower ω-6/ω-3 than those raised intensively on a concentrate feedlot diet or cereal grain rations (e.g., TMR).

Feeding concentrate-based diets to goats, regardless of the level of concentrate, is effective in increasing carcass weight, dressing percentage, rib-eye area, marbling score, total fat content, and producing chops with less off-flavour [87,88,89]. For example, it has been shown that supplementing East African goats with a concentrate diet (27% sunflower seed cake + 70% maize bran) increased carcass fat content [88]. Similarly, using a pelleted concentrate feed (26% cereal grains, 16% cottonseed meal, 30% cottonseed hulls, 20% dehydrated alfalfa, 5% pelletizing agent, and 3% minerals and vitamins) compared with a sorghum–Sudan grass hay diet increased carcass weight and internal fat in Boer crossbred goats and Spanish goats [89]. Concerning the FA compositions, goats fed concentrate feeds have higher percentages of oleic acid (C18:1) and ω-6 FAs, but lower percentages of ω-3 FAs, compared with goats fed no concentrate. It has been reported that feeding goats with a concentrate diet containing threefold oleic acid (C18:1) compared to the alfalfa hay diet increased C18:1 content in both intramuscular (marbling) and subcutaneous fat tissues [90]. In addition, when East African goats were supplemented with a concentrate diet (27% sunflower seed cake + 70% maize bran) the proportions of C18:1, unsaturated FAs, vaccenic acid (C18:1trans-11), and C18:2n-6 in the omental fat increased as compared to non-supplemented goats [91]. The increase in C18:1 in muscle or adipose tissues in response to higher concentration in the diet could be due to the increase in the Δ9 desaturase enzyme activities necessary for the de novo conversion of C18:0 to C18:1 [92,93]. Some studies have reported that the level of concentrate in the diet have minimal influence on fat content and FA composition of goat meat [84,91,94]. Increasing the concentrate level from 50 (41% dried-roll corn + 50% alfalfa hay) to 90% (77% dried-roll corn + 10% alfalfa hay) did not affect carcass fat content, colour traits, marbling score, and sensory characteristics of Boer crossbred goat meat [84]. It has been shown that the L* (lightness) and b* values of loin chops from concentrate-fed goats are lower than those fed the alfalfa diet; however, meat redness (a* values) did not differ [90].

#### 2.2.2. Effects of Feed Supplement Quantity, Quality, and Types on Chevon

Limited nutrient intake can significantly affect lean tissue accretion and minimise fat deposition. Restricting the amount of the daily feed intake has been found to decrease the daily weight gain of male Saannen kids and native goat breeds, but it did not affect the chemical, physical, or sensory quality measures of goat meat [95]. A greater average daily gain and less internal fat deposition have been reported after nutritional re-alimentation in Iranian native goats [96]. Changes in the quality of diet is another factor that can affect fat content, meat quality, and related gene expression in goats [97,98]. It has been shown that increasing energy levels of diet resulted in higher subcutaneous and intramuscular fat, and lower lean/fat ratios in Jordanian Black goats [99]. Changes in dietary energy and protein have altered the mRNA expression of some genes related to intramuscular fat and tenderness in LD and semimembranosus muscles of Hainan black goats [97]. The μ-calpain mRNA expression levels have been upregulated with increasing energy levels in the diet of goats, in contrast to the CAST mRNA expression levels [97]. Supplementation of diets with rumen undegradable protein and CLAs has decreased total fat content and increased cis-9, and trans-11 CLAs in the intramuscular fat of Kurdish goat kids [100].

The effect of the supplementation of diets with a different source of seed oils has been investigated by many researchers [19,39,101,102,103,104,105]. In all of the studies, supplementing diets with oils has not affected goat performance or their carcass traits, except for one study which reported that diets supplemented with 1% sunflower oil altered lipid accretion in different adipose tissues of local Bulgarian white goats [101]. The incorporation of 2% soybean oil [39] and 3% canola oil [19] into the goats’ diet has reduced kidney fat (peri-renal). The effects of dietary oils on the FA composition of chevon to a large extent depend on the FA compositions of oils. Supplementing goat diets with oils rich in C18:1 (e.g., canola oil) and C18:2n-6 (e.g., sunflower or soybean oils) mainly has resulted in an increase in C18:1 and CLAs in meat, respectively. Adding soybean or sunflower oils at 45 g/kg DM into a basal ration containing 40% berseem (*Trifolium alexanderinum*) hay and 60% concentrate (47% maize, 18% soybean meal, 12% de-oiled rice bran, and 20% chickpea chuni) has increased the proportion of PUFAs and cis-9, trans-11 CLA in the intramuscular and adipose tissues of Black Bengal goats [102]. The inclusion of 5% palm oil, a rich source of SFAs, into the diet has improved the ω-3/ω-6 ratios in the muscles of Kacang × Boer goats [103]. Adding a blended oil (80% canola oil + 20% palm oil) into a diet containing 47.5% palm oil by-products, 22% corn grain, 17% soybean meal, and 2% rice bran, has increased the proportion of C18:1, TVA, CLAs, C18:3n-3, and total ω-3 FAs in the supraspinatus muscle in goats [104]. Similarly, supplementing goat diets with sunflower and soybean oils has caused an increase in TVA and its isomerizing cis-9, trans-11 CLA in the longissimus muscle of goats [105]. These findings indicate that when goats are fed with oils rich in linoleic acid, more of the linoleic acid is isomerised to cis-9, trans-11 CLA and hydrogenated to C18:1 trans-11 in the rumen causing more deposition in the muscle tissues.

Supplementation of diets with oils rich in ALA (C18:3n-3) has caused an increase in ω-3 FAs in chevon. The incorporation of 3% canola oil, a good source of C18:3n-3 with a ratio of 2:1 of ω-6/ω-3, into the goats’ diet has enhanced 18:3n-3 in the longissimus lumborum muscle, liver, and kidney fats [19]. Replacing palm kernel cake with 10 and 20% whole linseed, as the source of C18:3n-3, has increased fat content, C18:3n-3, and total n-3 PUFAs in chevon [106]. Similarly, feeding fish oil, high in C20:5n-3 and C22:6n-3, has increased C20:5n-3 and C22:6n-3 proportions in the longissimus muscle fat of Mahabadi goats [39]. The inclusion of 1.3% linseed oil (>50% C18:3n-3) into the diet has increased the proportions of C18:0, C18:3n-3, C20:5n-3, C22:5n-3, and C22:6n-3 and has resulted in a decrease in ω-6/ω-3 ratios in the intramuscular fat of goats [105]. The oil supplementations of diets could also affect expression levels of genes associated with meat quality in goats [97]. Recently, it has been shown that a blend of linseed and palm oils (2:1) reduced both rumen biohydrogenation of C18:3n-3 and muscle oxidation of C18:3n-3 in Cashmere goats [107]. Diets supplemented with the blended oil, as compared to linseed alone, have reduced the relative abundance of *Pseudobutyrivibrio*, a bacterial species that hydrogenate dietary C18:3n-3 in the rumen, leading to a decrease in the ω-6/ω-3 ratio in goat meat. Furthermore, a decrease in the mRNA expression of CPT1β (a gene associated with fatty acid oxidation) and an increase in the mRNA expression of FADS1 and FADS2 (genes associated with ω-3 PUFA synthesis) have been discovered with the use of blended oils [107]. The existing data in the literature support the idea that dietary supplementation of oils rich in C18:3n-3 could be a beneficial strategy in improving the ω-3 FA content of chevon.

The inclusion of palm oil by-products, as a cheap feed with high fibre content, in the diet could also affect the FA composition of chevon [103,108,109]. Goats fed palm oil by-product-based diets (decanter cake and palm kernel cake) produced meat with a greater proportion of SFAs [103]. The higher proportion of SFAs in chevon of palm oil by-products-based diets could be explained by higher dietary fibre intake. Similarly, feeding diets with high fibre to Boer × Spanish male goats [90] and East African goats [91] has resulted in a greater SFA proportion in subcutaneous fat and minced meat, respectively. The inclusion of 21% palm kernel cake did not affect the physicochemical composition or nutraceutical compounds of chevon [109].

It is noteworthy that supplementing the diet with a high content of ω-3 FAs might increase lipid oxidation and may raise concerns about the impairment of sensory attributes of chevon. The inclusion of high doses of ω-3 FAs has produced meat with unusual odours, unpleasant flavours, and low overall appreciation of kid meat [110]. A positive association between lipid oxidation and the contents of ω-3 FAs in chevon has been reported [106]. Intriguingly, liver and longissimus lumborum muscle fats from canola oil-fed kids have contained fewer lipid oxidative substances as compared to those from palm oil-supplemented kids. It has been shown that canola oil effectively reduced lipid oxidation both in the blood and muscle tissue of goats [19].

Phenolic components have been evaluated to improve the physicochemical composition, FA profile, and sensory attributes of chevon [111,112,113,114,115,116]. It has been shown that supplementation of diets containing polyphenols positively improved the FA profile and reduced malondialdehyde concentration (a lipid oxidation substance) in chevon [117]. Pomegranate seed pulp, a cheap source of polyphenols, has decreased lipid oxidation, and improved colour stability in chevon [111]. Similarly, dietary green tea by-product (20 g/kg DM) has improved the redness and yellowness values and increased the proportions of MUFAs, PUFAs, and the PUFA/ SFA, ω-6 FAs and ω-3 FAs of chevon [118]. Dietary green tea by-products [118] and extract from olive mill water waste [117] have been shown to be promising in reducing the TBARS values of chevon. Similarly, replacing 15% olive leaf with alfalfa hay has improved the total antioxidant capacity in the meat, blood, and liver of Mahabadi goat kids [113]. Recently, it has been reported that the inclusion of condensed tannins from an *Acacia mearnsii* extract improved animal growth performance as well as the flavour, aroma, softness, and overall acceptance of crossbreed Boer goat meat [116]. Dietary supplementation with polyphenol extract powder from olive mill water waste has improved unsaturated FA/SFA ratios in the meat of Saanen goats [117]. Using a mix of dried olive leaves and dried *Stipa tenacissima* has improved the ω-6/ω-3 ratio of meat of Tunisian indigenous goats [112]. It has been shown that the inclusion of 15% olive leaf into the diets changed the fatty acid composition and improved ω-6/ω-3 ratios in both muscle and adipose tissues of Mahabadi goats [114]. Altogether, diets containing phenolic components seem to be positive agents in maintaining healthy FA composition in goat meat by reducing the potential for degradation and/or hydrogenation of long-chain and health-claimable PUFAs along the process of digestion and absorption in the gut.

### 2.3. Beef and Meat from Cattle

Beef is an end result of domesticated cattle (*Bos taurus*) production—although the point of harvest within an animal’s lifecycle will depend on its primary purpose, e.g., dairy, breeding, draft, or hobby [119]. It is recognised that the diet of cattle will contribute to the sensorial quality, nutritional value, and shelf life of its meat (beef) [7,120]. In response, much effort has been focused on the strategic modification of cattle diets to enhance the preferential attributes of the beef, as well as to maintain animal productivity and welfare [6,44,63,121]. Key elements of a balanced diet are macronutrients, such as carbohydrates, fat (energy), and protein, and various micronutrients, including minerals and vitamins [122]. These are often the target for modification in cattle diets, either by the supplementation or selection of rangeland, grazing, and concentrate-mixed basal diets. It should be remembered that these nutritional elements of a balanced diet should not be considered in isolation from each other.

Protein can be categorised as degradable (RDP) or non-degradable (RUP) depending on its passage through the rumen and its susceptibility to the proteolytic actions of ruminal microbes [100]. Good sources of degradable protein for cattle include quality pasture, silage, and grazing forages. Non-degradable protein is more often provided as a supplement to cattle raised on poor-quality rations and includes distillers’ grain, cereals, and other concentrates. Urea can also be used to improve the nitrogen and therefore protein content of poor-quality rations, although, as a non-protein source of dietary nitrogen, its inclusion into cattle diets must be strictly managed to avoid toxicity, growth suppression, and reduced muscle deposition [123]. Hence, it is important to match the protein content of a diet to the animal’s requirement for maintenance, growth, and muscle protein accretion.

The lean yield of a beef carcass informs its value, but it also contributes to post-mortem carcass temperature and pH declines that underpin many quality traits [124,125]. Beef tenderness, water-holding capacity, and colour stability attributes may be compromised if the rate of carcass temperature decline does not provide sufficient time for carcass acidification [126,127,128]. These carcasses are often identified using grade classifications and discounted by the industry [129]. Larger carcasses have greater resilience to cold-shortening and are likely to achieve appropriate temperature and pH declines—although fat coverage may deliver similar outcomes as a result of a comparable insulator effect. The level of protein in the diet will contribute to the growth rate of cattle and, ultimately, the hot carcass weight. Higher growth rates have also been shown to increase muscle protein turnover, promote a favourable ratio of connective tissue and proteolytic potential (enzymes) to lean muscle mass, and, therefore, enhance beef tenderness [130]. It should be noted that if dietary protein is excessive, the protein will be lost through excretion, and if dietary protein is insufficient, the protein and energy ratio will be affected, which in turn may affect cattle growth rates and muscle deposition.

Compared to rangeland and grazing systems, cattle raised on grain or concentrate-mixed rations have higher energy intakes, in the form of acetate, propionate, and butyrate—these being short-chain FAs. These systems may be combined and supplemented grazing cattle with energy-rich feed types to enhance the quality of their diet [131]. These supplements may be cereal grains and meals, by-products of biofuel production, distiller’s grain, molasses, and others [7,132,133]. Sufficient levels of energy in the diet are important for the metabolic function of cattle. Excess dietary energy can be stored within the muscle tissue as glycogen or in specific fat deposits as triglycerides [134]. The prevalence and concentration of these deposits will impact beef quality [60,64]. This proposes a pathway to manage beef quality via the modification of a diet’s energy contents.

**Figure 5:**
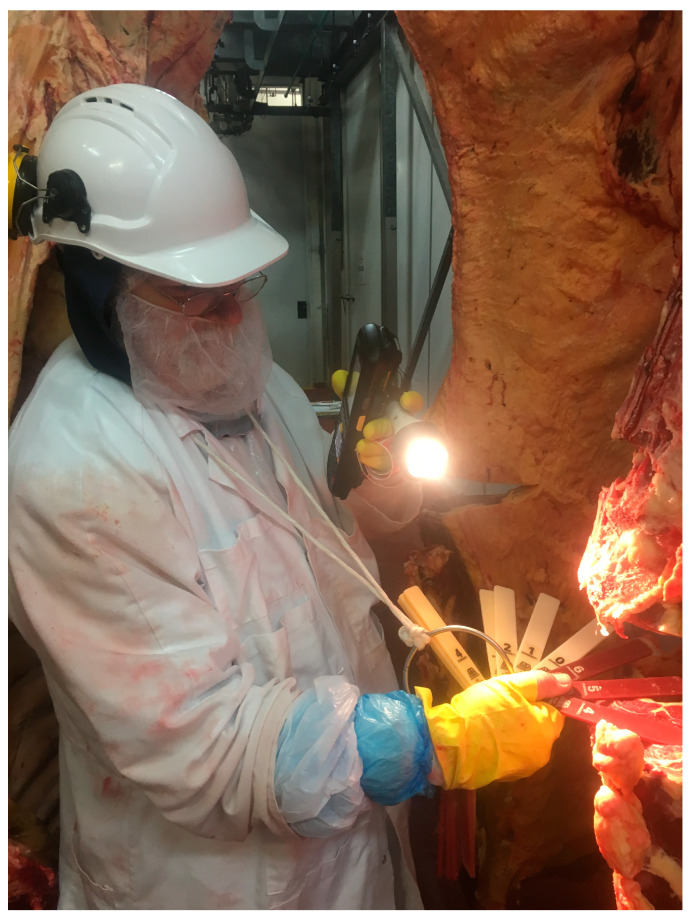
An Australian beef carcass being graded using colour chips to determine whether it is dark-cutting or not dark-cutting grade.

The concentration of glycogen in the muscles of cattle immediately before slaughter will determine the likelihood of dark cutting and the associated impact on beef retail potential [127,135]. Dark cutting refers to defective beef that is dry, firm, and dark beef as a result of suboptimal post-mortem acidification (Figure 5). The provision of high-energy feed types in the lead-up to slaughter has been shown to increase muscle glycogen reserves and, therefore, potentially reduce the incidence of dark cutting [135,136,137]. This outcome is subject to the severity of other factors that contribute to dark cutting, such as pre-slaughter stressors and post-slaughter carcass handling systems [138,139,140,141]. High-energy diets will also produce cattle with heavier carcass weights, higher levels of intramuscular fat, and increased fat coverage. These characteristics may attract a premium for some meat products (e.g., Wagyu) and drive improvements in eating quality—for example, the juiciness, palatability, and flavour profiles of beef are enhanced with higher levels of intramuscular fat [12,142,143]. However, in other instances, excessive fat deposition contributes to lower lean muscle yields, an increased need for carcass processing and trimming excess adipose or fat tissues, and leading to lower returns [7]. For these reasons, it is advised to premediate the end market for cattle when formulating the energy and protein content of their diet.

The energy content of a cattle diet can be enhanced with the provision of fats and lipids, with plant and marine oils, oilseeds, and fat supplements as examples of common feed types added to the basal diet of cattle [6,38,55]. Dietary fats can be categorised as protected or unprotected, depending on their susceptibility to biohydrogenation in the rumen. Protected fats are conditioned to limit their degradation in the rumen. Conditioning methods include the use of whole oilseeds, fat emulsification or encapsulation within proteins, chemical interventions to form calcium soaps, amides, and otherwise inferred protection against rumen degradation [6,144]. Instead, unprotected fats are subjected to ruminal degradation, whereby PUFAs are hydrogenated into SFAs and intermediatory MUFAs as a result of the lipase activity of ruminal microbes. Biohydrogenation is not comprehensive, with a portion of unprotected fats bypassing degradation in the rumen and available for absorption and deposition in the muscle [145]. The proportion of unprotected fats that bypass degradation in the rumen is increased as dietary levels increase; however, excessive dietary fat levels (>6%) can compromise rumen function and cattle productivity. Regardless, the provision of dietary fat of either category will have implications on beef quality, nutritional value, and sensorial attributes.

**Figure 6:**
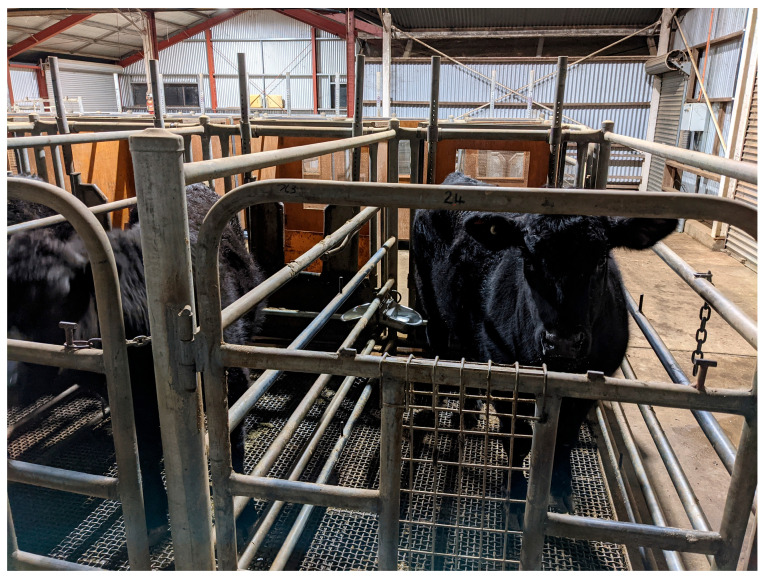
An Australian Angus steer held within an individual metabolic crate during a feeding study comparing the effects of different types of supplementary fats on meat quality.

Through dietary modifications, the concentration of health-claimable FAs in beef can be increased [146]. The inclusion of algae, fish oil, flaxseed, and canola meal into cattle rations have been shown to increase the concentration of EPA, DPA, DHA, and total ω-3 FAs in the beef [6,18,147]. These FAs contribute to a more balanced ω-6/ω-3 FA ratio in ruminant products, as this is somewhat skewed in the typical modern diet [148]. Odd- and branched-chain fatty acids may offer health benefits to consumers [73], and their concentrations may be altered via nutritional interventions. For example, research has demonstrated that cattle fed flaxseed produces beef with higher concentrations of these FAs (specifically iso-17:0 and anteiso-17:0) than cattle fed cottonseed within the same mixed ration [73]. Enhancements to FA profiles are not limited to the provision of supplements, with cattle grazing pastures and fed silage producing beef with higher concentrations of ω-3 FAs when compared to cattle fed concentrate-mixed rations, rich in cereal grains, that produce beef with higher concentrations of ω-6 FAs [6,149]. This demonstrates the value of understanding the effect of basal diet on beef quality; in some instances, further supplementation may not be necessary (Figure 6).

Beef flavour (aroma and taste) is derived from the thermal reaction of non-volatile FAs upon cooking, with the type and concentration of volatiles released being dependent on the initial FA profile [150,151]. This principle can be illustrated by cattle fed fish oil supplement producing beef with an unacceptable ‘fishy’ aroma and taste, while grass-fed cattle produce beef with a characteristic ‘grassy’ odour [152,153]. PUFAs are also highly susceptible to oxidation, which, in sufficient concentrations and inducive storage conditions, can contribute to the generation of volatiles associated with rancidity and malodourous beef [14,50]. Furthermore, the free radicals and secondary metabolites generated from lipid oxidation are also implicated in beef colour deterioration upon retail display [154]. Consequently, beef with higher PUFA concentrations may be at increased risk of consumer rejection and reduced retail potential than beef with lower concentrations. This point notwithstanding, research has found it possible to mitigate these outcomes with the appropriate inclusion of antioxidants in the diet of cattle.

Rangeland and grazing production systems offer substantial basal concentrations of vitamin E (α-tocopherol), although the stage of plant maturity will impact concentrations [14]. Large ruminants, such as beef cattle, maintained indoors on concentrate diets or feedlots and of more than 9–12 months old should be provided with adequate antioxidants, when their diets lack in green pasture or silage materials. Cattle fed concentrate-mixed rations must instead source vitamin E from supplementation to achieve the recommended daily intake of 15–40 mg per kg dry matter [155]. Natural and synthetic forms of vitamin E can be supplemented in cattle feed, but free tocopherol is more effective in terms of transitioning from the diet into muscular systems than tocopheryl acetate. Sources of natural vitamin E include fresh pasture, fodder crops, legumes (e.g., lucerne), silage, yeasts, and specialised forage plants. It has been reported that a vitamin E concentration of 3.30 mg per kg of beef is sufficient under commercial conditions [156], although levels down to 0.90 mg per kg have also been reported as being sufficient for short-term chilled storage durations. This is because, in the muscle, vitamin E acts as a free radical scavenger, reducing the capacity for lipid oxidation and, doing so, preserves fatty acids and proteins against deterioration [157]. As a result, vitamin E helps stabilise the nutritional value, retail colour, and flavour properties of beef [14,158]. Other antioxidants infer similar benefits to beef quality, and while they can be provided within cattle diets, their contributions are relatively minor in comparison to that of vitamin E. These include vitamin C, carotenoids, selenium, and polyphenol compounds that are often available from the same dietary sources as listed for vitamin E [14,159]. They may, however, offer secondary benefits to beef quality—for example, dietary tannins have been shown to help minimise biohydrogenation in the rumen of PUFAs to increase their availability for absorption [160].

## 3. Nutritional Effects on the Meat from Alpacas and Llamas

South American camelids, including llamas (*Lama glasma*) and alpacas (*Vicunga pacos*), are not primarily used for meat production but instead used for fibre production or as a sumpter animal. These functions notwithstanding, llama and alpaca meat are important food resources, consumed by many around the globe, especially the rural communities of the Andean highlands. Much effort has also been applied to determine and improve the quality of South American camelid meat to diversify and grow its market share [161,162,163,164]. Fundamental to this outcome is the replacement of traditional native pasture feed base with improved pasture mixes and concentrate supplementation.

South American camelid meat is characteristically lean, with low levels of intramuscular fat (0.5–3.0%) and high levels of protein, vitamins, and minerals (e.g., vitamin B, zinc, iron, etc.) [165]. Research has demonstrated that the supplementation of llamas with sorghum–wheat bran concentrate increased the dietary nutritional value of native pastures and resulted in higher intramuscular fat levels than supplementation with lucerne hay [166]. The fat levels of alpaca meat have likewise been increased with advancements to basal diet nutritional values—evident by comparisons of animals raised on improved pastures in Australia to those raised on unimproved traditional pastures of lower nutritional value. The supplementation of grain to alpacas grazing improved pastures, and increased growth rates and intramuscular fat levels, but not to the extent expected by researchers who suggested more substantive improvements if the quality of the basal diet was reduced [167,168]. Evaluation of alpaca meat quality traits found no seasonal effect on intramuscular fat content, ultimate pH, cooking losses, and tenderness values, although pasture energy, crude protein, and neutral digestible fibre levels varied significantly between the seasons [163]. The impact of nutrition on the eating quality traits of camelids is of interest because of their comparative toughness when compared to other ruminant meats.

The tenderness of llama and alpaca meat is comparable, having a Warner–Bratzler shear force range of 40–95 N, depending on animal age, muscle, and post-mortem intervention [165]. The comparison of feeding systems has only marginal effects on tenderness; however, the selection of feeding systems impacts the FA composition and nutritional value of South American camelid meat. The levels of ω-3 FAs in the meat are highest in llamas and alpacas fed pasture-only diets, and these levels decline when the animal is supplemented with a grain-based concentrate. Increases to the health-claimable ω-3 FAs (EPA and DHA), as well as total PUFAs, have been attributed to extensive rearing on high-quality pastures. Indeed, the low levels of intramuscular fat and high levels of vitamin E in South American camelids grazing in this system could act to preserve this fraction in the meat until consumption—as demonstrated by its low susceptibility to peroxidation [169]. These same nutritional outcomes also contribute to the colour stability of llama and alpaca meat, a concept to note when selecting production systems to enhance South American camelid meat quality and retail potential.

## 4. Nutritional Effects on the Milk from Dairy Cows, Goats, and Buffalo

**Figure 7:**
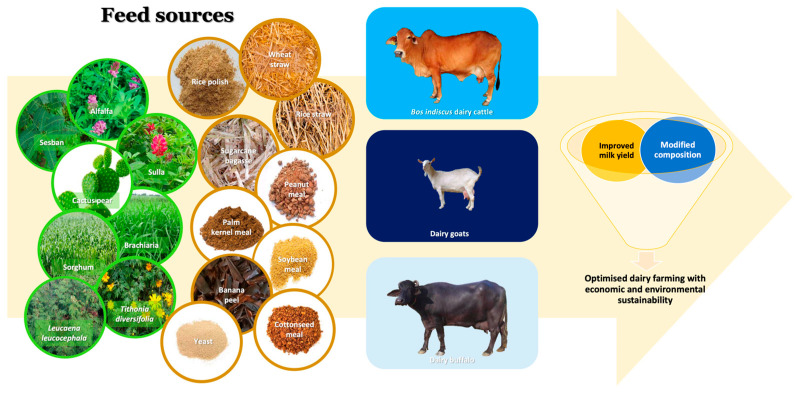
Some examples of feed sources utilised in farming systems of tropical regions around the world for meat and milk production with economic viability and environmental sustainability.

Managing nutrient requirements for dairy animals is an important consideration for optimal animal performance and product quality. Feed and feeding strategies in dairy animals play a significant role in altering their milk composition and properties. In the processing of value-added dairy products, the composition, nutrition, and organoleptic and technological properties of raw milk are a vital concern. Often, raw milk quality attributes are impacted by dairy cow management and nutrition, cow genetics, and processing technologies [170]. Among these strategies, managing dairy animals and their feeds is a crucial step in enhancing the quality and composition of milk intended for better processability and nutritional profile. Furthermore, the utilization of various feed sources and efficient feeding management in dairy animals are also widely accepted as a strategy for enhanced sustainable animal production (Figure 7).

Several studies have been conducted to elucidate the effect of feeding systems, feed types, and feed composition on milk yield and milk composition in *Bos indicus* dairy cattle, goats, and water buffalo. Moreover, at present, interest in altering milk nutritional components for the better well-being of consumers is growing. The inclusion of non-conventional feedstuffs into ruminant diets is also gaining increased popularity as a strategy to combat climate change, particularly increased drought conditions associated with the diminishing nutrient value of feeds. Often, supplementary sources (e.g., neem cake, castor oil cake, karanja cake, rubber seed meal, guar meal, and palm kernel cake) are incorporated into dairy cattle rations to enhance crude protein content. British and European dairy cattle (*Bos taurus*) are reared under temperate or Mediterranean climates where they consume different pasture, fodders, and specialised forages as mixtures or single strands, which is different from dairy cattle (*Bos indicus*) raised in tropics exposed to different climatic conditions and feeds. There are various feedstuffs and supplementations fed to dairy animals around the world based on their availability and affordability. The types and composition of some feed sources used for cattle is presented in Table 2. The following sections provide an overview of various diets and feeding strategies adopted for milk (dairy) producing ruminants such as cattle (*Bos indicus* and *Bos taurus*), goats (*Capra hircus*), and water buffalo (*Bubalus bubalis*) reared in tropical regions focusing on milk fat content, fatty acid composition, and sensory attributes (e.g., taste and flavour).

### 4.1. Bos Indicus—Milk Yield and Composition

To understand the effect of diet on milk production and its composition in *Bos indicus*, several studies have been conducted to evaluate the effect of changing the diet from a system combining TMR and pasture to various dietary inclusions. In tropical regions, the provision of numerous grasses and foliage types that are predominantly available under different farming systems is commonly practised. Thus, evaluating and comparing the potential of varying types of grasses and foliage on milk yield and composition is crucial. The provision of elephant grass silage, together with concentrates, resulted in lower milk yield, protein content, and SFAs, and higher UFAs and CLAs when compared to supplementation with briquette from *Brachiaria* and sugarcane [171]. However, the supplementation of *Brachiaria* pasture, together with mineral supplements (nitrogenous and mineral salt) and a concentrate consisting of corn and soybean meal, resulted in no changes in milk constituents in crossbred Zebu cows [172]. A study by Peniche-González et al. [173] evaluated the provision of restricted concentrate and forage consisting of either Leucaena (*Leucaena leucocephala*) or Stargrass (*Cynodon nlemfuensis*) into crossbred Zebu diets and observed no differences in milk yield or gross composition. Additionally, the provision of Leucaena, together with sorghum grain, in the silvopastoral system has been shown to be a viable alternative for expensive conventional concentrate diets provided in Stargrass monoculture [174]. The incorporation of dried Sesbania sesban leaves into the basal diet (control, 0%) in varying levels (1.25, 2 and 2.75 kg/day) into crossbred Zebu (Arado) diets showed an increase in milk yield when *Sesbania sesban* leaves were supplemented at 2.00 and 2.75 kg/day and compared to the non-supplemented diets [175]. The effects of including cactus pear in association with sorghum silage or elephant grass into crossbred Zebu diets were evaluated by Borges et al. [176] and found no alterations to the milk yield by providing cactus pear with sorghum or elephant grass silage.

Provisions of energy supplements, e.g., highly fermentable (sugarcane molasses or fresh citrus pulp) and starch-based carbohydrates (sorghum grain or rice polishing) into Zebu cows feeding on Leucaena foliage resulted in no influence on gross milk composition; however, milk yield was increased when sorghum and rice polishing were supplemented [177]. In another study, feeding two different energy supplements (corn grain and wheat bran) with two types of straw sources (wheat and oat straw) to Sahiwal cows resulted in no influence on milk content or the gross milk composition [178]. The inclusion of palm kernel cake at 150 g/kg dry matter into the crossbreed Zebu ration did not alter the milk production or composition [179]. However, the inclusion of Crambe meal into the sorghum silage diets of Zebu cows increased the milk production without altering the gross milk composition [180]. Replacing peanut cake with soybean meal for crossbred Zebu diets, Dias et al. [181] reported no differences in milk yield, protein, fat, lactose, and total solids concentrations between the cows provided soybean meal or peanut cake rations. Alves et al. [182] evaluated the effect of replacing soybean meal with increasing levels of cottonseed meal in the concentrate diets for crossbred Gyr dairy cows and reported no influences by cottonseed meal in the diet on average milk yield and milk yield corrected for milk fat content. The substitution of soybean meal with a yeast-derived protein in the crossbred Gyr dairy cows’ ration under grazing (Guinea Grass, *Panicum maximum* cv. Mombaça) led to a decreasing milk yield and solid contents due to decrease in the neutral detergent fibre intake, without influencing the total feed intake [183]. Feeding of maize silage diets to dairy cattle has increased the milk fat percentage but the supplementation of fibrolytic enzymes, glycerol supplementation, and dietary tannins has decreased the milk fat component.

### 4.2. Bos Taurus—Milk Yield and Composition

The knowledge concerning the impact of feeding *Bos taurus* dairy cows on milk yield, composition, and quality is continuously evolving with the advancement of research output in relation to various forage types, their composition, conservation strategies, cow welfare, environment (climate and diseases), genotype, parity, stage of lactation, pregnancy, and husbandry practices. The producer is paid not only for the quantity of milk produced but also for the milk constituents such as fat and solids-not-fat (SNFs). There is a variation in the volume and composition of milk production among different *Bos taurus* breeds such as Holstein Friesian, Jersey, Ayrshire, Brown Swiss, Guernsey, etc. On average, even within a herd production, variation could exist up to ~40% due to heritability characteristics. The productivity of a cow gradually increases to around its fifth lactation (parity) due to increasing amounts of secretory tissues being deposited in the mammary system as the calf birth number increases. However, total solids tend to be higher during early parities than in late parities in many *Bos taurus* breeds. Milk production is closely related to the quantity and nutritive characteristics of feed consumed. The impact of nutritional effects could be both via short-term (daily) nutrient intake and also due to carry-over effects influenced by changes to the body condition of dairy cows. Both pre-partum and post-partum nutrition could have a major influence on milk yield as well as the milk composition of cows. It is known that during late lactation and the dry period, most of the fat reserves are deposited and will provide sufficient body reserves for early lactation (first 10–12 weeks) during which the cow’s metabolic demand cannot be fulfilled solely by the diet consumed.

The level of nutrition during post-partum should be aimed at maximising milk production, while simultaneously maintaining its quality standards. It is important to avoid extreme mobilisation of body fat reserves as it can affect cow health as well as future fertility and conception rate. High-quality forage supplementation to dairy cows has a positive influence on their milk yield. The addition of concentrate supplements to animal diets (supplementation) appears to result in a consistent response in milk yield throughout the lactation period of dairy cows. Moreover, the interaction between nutritional and seasonal effects on milk production and composition is difficult to separate in dairy cows. Factors such as the amount of roughage, the ratio of forage to concentrate, the carbohydrate composition of the concentrates, types and amount of lipids present in the diet, and feeding frequency have a marked influence on the fat content in milk.

**Figure 8:**
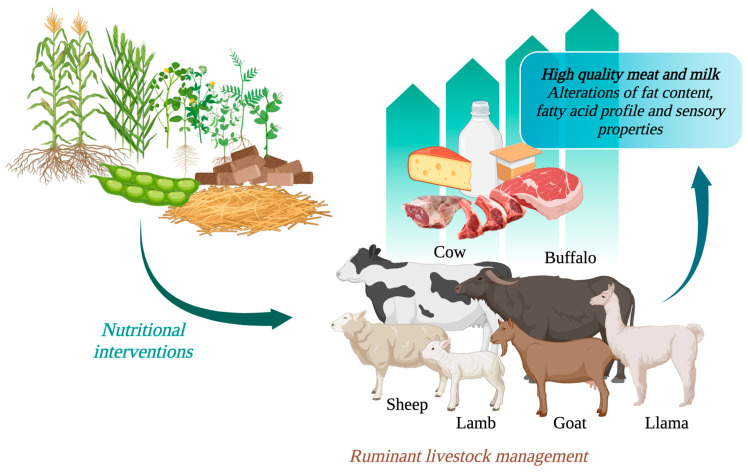
A holistic approach of forages, roughage, and supplement selection for feeding domesticated ruminants, which can optimise the quality and nutritional value of meat and milk products.

Optimal feeding for dairy cows has been rigorously researched with findings having contributed to an improved understanding regarding the relationship between feed components on milk production and concentration of milk constituents (Figure 8). The quality of raw milk and processed dairy products is closely associated with on-farm management factors and feeding strategies. For example, grazing Holstein Friesian cows offered corn grain and canola meal showed a positive linear relationship between supplementation level and milk yield and protein content, and a negative linear relationship to milk fat content. Some of the differences in milk quality observed between the concentrate supplementation levels were due to intake differences, in terms of basal pasture diet and supplements [184]. Relating to this, the time of grazing of a ryegrass pasture was found to impact milk quality, as a result of changes to the herbage nutrient supply. Specifically, it has been reported that morning grazing will result in increased concentrations of rumenic acid and PUFAs in the milk, compared to cows allowed to graze only in the afternoon [185]. Holstein Friesian cows grazing perennial ryegrass pastures and fed a simple mix of pasture silage and grain had comparable effects on milk quality as conventional grain supplementation at milking (in the parlour). Milk fat yield was slightly improved with the use of an isoenergetic ration, that contains lucerne, corn grain, and silage [186]. It was noted with Italian Brown cows grazing on mountain pasture that the higher supplementation level (4.8 kg organic matter/day) resulted in improved milk yield and cheesemaking properties when compared to a low level (1.6 kg organic matter/day) [187]. Summer transhumance (i.e., moves from permanent farms in the valleys to temporary farms in highland areas) is a practice in mountainous areas to exploit the Alpine pastures [188]. However, milk volume was lowered when cows were moved to Alpine pastures during summer as reported by Bergamaschi et al. [189]. The latter outcome is probably an effect of the nutrient imbalance of diet with the movement to the highlands and this was recovered when the cows were back in permanent farms. Nevertheless, milk quality was affected with alpine transhumance, where Koczura et al. [190] observed an increase in somatic cell count and milk fat content immediately after moving cows to Alpine pastures.

The type and diversity of the forage will also influence the milk FA profile. Feeding of pasture at the early growth stage increased the content of trans-11-18:1 and cis-9, trans-11-18:2 in milk when compared with feeding concentrate and hay 65:35, respectively. However, forage did not affect the proportion of butyric acid and short-chain FAs as compared to feeding concentrate and hay diets [191]. Milk lipolysis, which hydrolyses the milk fat into free FAs and causes a rancid flavour in milk, is undesirable for consumers or dairy processors, especially for milk destined for processing or consumption. When the cows were provided with concentrates and a hay- or corn-silage-based diet, as compared to a ryegrass-silage-based diet, lipolysis values of milk were reported to be lowered [191]. This is because milk yield has a negative relationship to lipolysis and higher lipolysis can occur with ryegrass-silage feeding. Shingfield et al. [192] evaluated the effect of forage type and level of concentrate in the diet of British Holstein Friesian cow milk’s FA composition. In the latter study, authors reported that corn silage inclusion, when compared to grass silage, resulted in higher proportions of trans-10 C18:1 and lower proportions of C18:0 (stearic acid) and CLAs in milk fat. The incorporation of flaxseed supplementation into dairy cows’ diets is a viable strategy to provide a higher level of energy and ω-3 FAs, where Isenberg et al. [193] observed that supplementing Jersey cows with ground flaxseed did not alter the milk yield and components, but increased ω-3 FA concentration in milk.

Providing TMR to dairy cows enables more efficient milk production, as a result of optimised dry matter intake and daily milk yield [194]. Cheese made of milk obtained from farms providing TMR consisting of maize silage was characterised by an intense smell of sour milk and a firmer texture than milk derived from farms without using silage-based TMR (e.g., only hay and commercially compounded feed) [195]. This is because, the farms using TMR that contain maize silage fed lower proportions of CLAs, linolenic acid, and TVA to their animals and, thereby, modified the susceptibility of the resulting cheese to oxidation and lipolysis. However, the provision of silage-based TMR containing hay, maize, grass silage, imported alfalfa hay, and concentrates (commercial compound feed or a mix of cereals, e.g., maize grain, soybean meal, and dry beet pulp) compared with feeding year-round on the farm’s meadow hay and commercial compound feed did not affect the milk coagulation, firmness, or syneresis [196].

In an Irish study by Boiani et al. [197], feeding spring-calving Friesian cows with a TMR diet indoors compared to outdoors on pasture (perennial ryegrass or a combination of perennial ryegrass and white clover), resulted in higher concentrations of citrate content in milk. However, a Danish study investigating the variation in feed differences associated with two organic farms and a conventional farm observed no increase in citrate content in milk in connection with pasture feeding in organic farms [198]. Similarly, Chen et al. [199] reported that citrate content did not vary with seasons in bulk milk in year-round calving Holstein cows in the UK. The inclusion of wheat straw into the TMR diet of Holstein dairy cows in the first 21 days after calving reduced the feed intake and resulted in decreased milk yield [200]. Feeding Swedish Holstein and Swedish Red cows with a by-products-based diet (e.g., sugar beet pulp, rapeseed meal, distiller’s grain, and wheat bran), together with highly digestible grass-clover silage ad libitum, found no differences in overall energy-corrected milk yield and milk fatty acids [201]. The effect of incorporating ground corncobs as a fibre source in TMR on feed intake, milk yield, and composition in tropical lactating crossbred Holstein cows was evaluated by Wachirapakorn et al. [202]. This study reported that ground corncobs can replace rice straw in TMR without altering the milk composition, although, milk yield was highest when the ground corncobs to rice straw ratio were 100:0 and 82.5:17.5, compared to 67.5:32.5, and 50:50.

Feeding various pomace (e.g., olive and grape) rich in biologically active compounds (e.g., polyphenols) and high crude fibre content derived from a solid by-product of olive oil separation and the wine industry, respectively, provide opportunities to improve the nutritional and sensory attributes of milk. The dietary inclusion of dried olive pomace at 10% of dry matter in feed rations decreased palmitic, stearic, and linoleic acid concentrations while increasing oleic acid concentrations in milk compared to the cows fed without dietary supplementation. However, milk yield and composition (fat, casein, lactose, urea, and somatic cell count), except for higher protein contents, were not affected by providing supplementation [203]. Dietary grape pomace supplementation to lactating cows increases the concentration of PUFAs and has been proven to contribute a pleasant aroma and sensory properties to the resulting cheese [204]. The above findings indicate that the inclusion of pomaces in dairy cattle feed can improve the nutritional profile and functionality of milk.

Feeding rumen-protected lysine to Holstein cows to increase lactation performance found that energy-corrected milk yield, fat, true protein, casein, and lactose concentration were increased in the post-partum period when cows were supplemented with rumen-protected lysine in the pre-partum period [205]. Blends of eugenol, coriander essential oil, and geranyl acetate included in the TMR of Holstein cows were compared as modifiers of rumen fermentation to decrease methane production and Elcoso et al. [206] reported an increased energy-corrected milk yield after four weeks of providing the supplementation. The information reported above in both dairy cattle sections (*Bos indicus* and *Bos taurus*) indicates that the alternative forage feeding strategies or supplementation of dairy cattle with cereal grains and protein meals diets are viable options for altered milk production and milk composition in dairy cattle of different breeds.

### 4.3. Sheep—Milk Yield and Composition

Non-bovine milk such as sheep and goat milk is becoming popular as an alternative milk source for cow milk, mainly due to the convenience in rearing these animals and the nutritious profile of their milk compared to cows [207]. Fatty acids, immunoglobulin and non-immune proteins, and prebiotics and probiotics in sheep milk provide comparatively greater functional and nutritional properties than milk from other ruminants [208]. Sheep milk contains bioactive peptides that can exhibit antibacterial, antiviral, and anti-inflammatory properties in humans [209]. Moreover, Flis and Molik [209] reviewed that sheep milk contains various other bioactive substances such as CLAs and orotic acid, which have the ability to prevent the occurrence of type-2 diabetes, Alzheimer’s disease, and cancer.

In most farming systems, rearing sheep is mainly for meat and wool, but not for milk production and, thus, sheep milk is an uncommon food commodity in the vast majority of developing countries. Sheep milk contains a greater concentration of UFAs, calcium, phosphorus, iron, and magnesium than cow milk [210]. Nevertheless, in the global market, sheep milk is gaining considerable popularity as a superfood, which has numerous health-promoting and nutritional benefits due to its rich composition. Because of this growing interest, various attempts have been made to manipulate the composition and properties of sheep milk through alterations in the feeding and management strategies of dairy sheep.

The milk yield and composition of traditionally grazed Araucana creole ewes were evaluated by Inostroza et al. [211]. They reported lower milk yield from Araucana creole ewes compared to other dairy sheep. A higher content of oleic acid was observed at an early stage of lactation compared to later stages of lactation and the highest level of CLAs was reported on the 60th day of lactation. Soják et al. [212] analysed the milk from three types of breeds (e.g., Tsigai, Improved Valachian, and Lacaune) in natural pasture feeding and reported that the CLA content in ewe milk differed significantly (fivefold) among individual animals. Lacaune breed yielded a greater amount of milk compared to other two breeds. The authors also observed an increase in milk yield, an increase of SFAs and a decrease of UFAs, however, oleic, and linoleic acid variation was insignificant. Zhang et al. [213] fed East Friesian and Lacunae crossbred ewes with supplements rich in linoleic and linolenic FAs, 260 g/kg sunflower seed or 300 g/kg flaxseed and they found that the latter group yielded more milk to the former when compared with a non-supplemented control group. Gallega sheep and Cashmere goats grazing on grass-dominated, gorse (*Ulex gallii*)-dominated, or heather (*Ericaceae*)-dominated heathland were compared by Osoro et al. [214]. They reported that both species adapt feeding preferences to season and forage availability, however, goats consumed more heather/gorse and less herbaceous plants than sheep.

In Mediterranean regions, dairy sheep are reared mainly under extensive and semi-extensive systems, whereas the overall management practices vary with the breed, production system and country. Under these farming systems, sheep get access to native pastures, forage crops or stubbles, and receive supplementation of commercial concentrates, grains, hay or silages [215]. The nutritional status of the ewe is closely related to the composition of sheep milk as reviewed by Pulina et al. [216], where the ewe’s net energy balance, dietary NDF content and supplementation with rumen-protected or unprotected marine and vegetable oils directly alter the milk fat content. Moreover, through feeding strategies, it is possible to alter the milk FA composition of sheep milk, where mostly aiming to increase the health-beneficial FA content, e.g., CLAs and ω-3 FAs.

The use of agro-industrial by-products as animal feed ingredients has attracted much attention, mainly due to its nutritional value and possibilities in reducing the cost associated with traditional feed ingredients that are expensive. In this context, Hilali et al. [217] evaluated the possibilities and potentials of incorporating several by-products such as molasses, sugar beet pulp, or cottonseed cake into the Awassi dairy sheep’s diet in the Middle East. The authors reported that the use of cottonseed cake enhanced milk yield, MUFAs and PUFAs. However, the use of molasses in sheep’s diet reduced milk yield and increased C6:0, C8:0, and C10:0. In another study, the provision of ensiled olive cake supplementation to purebred Chios sheep by Symeou et al. [218] reported a reduction of SFAs and increment of UFAs, MUFAs and CLAs. However, the milk yield or fat content of the ewe’s milk was not affected with ensiled olive cake supplementation.

Supplementation of sunflower seeds (106 g/kg DM) and linseeds (105 g/kg DM) with concentrate to Sarda ewes by Cabiddu et al. [219] reported an increment of total C18:2 non-conjugated dienes, C18:1 trans 9 and total trans C18:1 compared to the controlled grazing group, without supplementation. Furthermore, increased PUFA content was also observed in an oilseed-supplemented group than in a non-supplemented group. Essential oils, minerals and vitamin supplementation to dairy ewes can reduce the somatic cell counts, proving that appropriate feeding management of dairy sheep herds enables them to obtain high-quality milk with an enhanced nutritional profile [220].

In addition to the feeding and nutritional factors, Peana et al. [221] showed the effect of winter and spring meteorological conditions on milk production of grazing dairy sheep under Mediterranean farming systems. They found milk yield is closely associated with meteorological factors, where adverse temperature changes in spring and winter decrease the milk yield since dairy ewes are more sensitive to low temperatures. The authors proposed several remedies and suggestions for minimizing the adverse thermal conditions on the performances of sheep by providing an adequate number of grazing hours/times, proper type/amount of feed supplementation, pasture improvement, use of the shelter, and adequate ventilation. The season is directly associated with the pasture quality, where Altomonte et al. [222] showed the effects of the pasture season on the nutritional and volatile compounds of Massese sheep milk and processed dairy products such as yoghurt and cheese. The authors found that milk derived during the spring season had higher dry matter, calcium, cis-9, cis-12, cis-15 C18:3, and trans-11 C18:1 FAs, both in milk and dairy products. Further, cheese made from spring milk had a greater content of alcohols and esters, deriving alcoholic and floral flavour attributes.

### 4.4. Goat—Milk Yield and Composition

Goats are well known for their ability to graze and browse for a wide range of forages including plants with thorns and spines. Further, goats are well-adapted to live in harsh environments because of their low body mass, low metabolic requirements, skilful foraging behaviour and physiological adaptive features [223]. They ingest a wide array of browse species of pasture, fodders, and shrubs that are generally rich in tannins, as goats’ ability to utilise plants with higher condensed tannins provide them a competitive advantage. However, despite their high tolerance for tannins, ranging goats forage to minimise the consumption of tannins through altered bite rate, bout number, and bout length [224]. Goats are mainly reared in two production systems, i.e., kept in pasture or zero-grazing in confinement with concentrate feeding. In these two major production systems, the type of feeding and feeding strategies vary to a certain degree that will exert a greater influence on milk yield and composition. Alpine goat milk obtained from free-range grazing differed from milk derived from goats raised in permanent confinement, where higher total polyphenol compounds and antioxidant capacity were observed from goats from free-range grazing by Chávez-Servín et al. [225]. The comparison of Nubian and Granadina goats on grazing in arid rangeland during the rainy period by Mellado et al. [226] confirmed that Granadina goats consumed more shrubs than Nubian goats, while forbs consumption was higher in Nubian goats compared with Granadina. Because goats are highly selective feeders when they are in free-range systems, the dietary selection of feed materials varies (e.g., forbs 8–64% and browse 35–88%), while the dry matter digestibility of forage ranges from 44–65% [227]. Thus, there is growing interest in understanding and comparing the effect of varying types and quality of feeds on the milk quality characteristics of dairy goats. This is partly also due to goat milk providing basic nutrition to rural subsistence farmers as well as high-end consumers with sophisticated sensory, nutritional, and medicinal aspects [228].

Novel knowledge is constantly emerging by investigating the inclusion of various types and levels of feed ingredients, thus creating topical interest in evaluating the case for various goats. The feeding of goats (e.g., Alpine and Saanen) differing in the proportion of concentrates (30% or 60% concentrate) by Giger-Reverdin et al. [229] showed higher milk yield when goats were fed diets with 60% concentrate than 30%, but with lower fat and protein. This difference was attributed to differences in the nutritive values of feed since goats received more energy and nitrogen when they were fed high proportions of concentrates. Furthermore, the authors concluded that including diets with higher concentrates affected the feeding behaviour (i.e., eating more rapidly) and modified rumen parameters (e.g., decrease in pH, acetate/propionate ratio and increase in total VFAs, ammonia, and soluble carbohydrate concentrations).

Interest in altering milk nutritional components for the better well-being of consumers is ever-growing. Increasing levels of beneficial fatty acids, bioactive peptides, and antioxidant compounds in goat milk have, therefore, received considerable attention as reported by Verma et al. [230]. Thus, improving the fatty acid profile has been focused on the thorough modification of the metabolism of ruminal microbiota through nutrition and feeding [231], as well as providing protected fat against rumen metabolism [232]. The inclusion of lentisk foliage (*Pistacia lentiscus* L.) in Damascus dairy goats’ diets increased the ω-3 fatty acid content in milk [233]. PUFAs are known to provide various health benefits [234] and thus have gained attention to improve the PUFA content in milk through various strategies to improve the nutritional benefits to humans. Higher PUFA levels were observed when Granadina goats were fed with rumen-protected fat. However, milk yield, total solids, protein, fat, and lactose concentration did not differ [232]. PUFA and ω-3 FA content were higher on Girgentana goats fed with Sulla (*Hedysarum coronarium* L.) fresh forage [235]. Sulla contains high protein content, structural carbohydrates, total polyphenol, and non-tannic polyphenol compounds, as well as a moderate amount of condensed tannins [236]. The inclusion of Sulla fresh forages to Girgentana goats’ diets increased milk total protein, casein, total polyphenols, and free polyphenols contents, compared to feeding a diet of hay with barley meal [237]. Furthermore, Girgentana goats fed with Sulla fresh forage and 800 g/day of barley increased the energy intake, dry matter intake, crude protein intake, and milk yield compared to goats fed with mixed hay and 800 g/day of barley [235]. Examining the CLA content of goat milk by Tsiplakou et al. [238] revealed significant increase in CLA content during April–May, when goats are shifted from hay- and concentrate-based diets to green pasture-based diets. Further, the authors found that CLA content gradually decreased with the increasing maturity of grasses and advancement with lactation.

Red Syrian goats were fed seven different forage species (*Festuca arundinacea*, *Hordeum vulgare*, *Triticosecale*, *Pisum sativum*, *Trifolium alexandrinum*, *Vicia sativa*, and *Vicia faba minor*) to evaluate the antioxidant capacity, polyphenol, and fatty acid concentrations in cheese manufactured from forage-fed goat milk [239]. The authors reported that the species of forage influenced the antioxidant capacity, polyphenol, and fatty acid contents, by demonstrating the importance of type and species of forage in manipulating the composition of milk. These indicate that the proper planning of ad libitum feeding of selected forage species would confer desired nutritional benefits to the consumers of goat milk through its modified content of bioactive compounds. Evaluating the effect of two feed systems on the antioxidant content of soft cheese made from French Alpine goats, Hilario et al. [240] concluded that the feeding system modified the antioxidant profile of the resulting cheese through its raw milk. The same authors also observed that goats grazing outdoors resulted in a more favourable concentration of bioactive antioxidant compounds (e.g., polyphenol, hydroxycinnamic acids, and flavonoid) than indoor fed goats. These components may transfer from forages, where Sulla fresh forage fed to Girgentana goats demonstrated an increased antioxidant activity due to its secondary free polyphenol compounds [237]. Milk derived from goats fed with green borage plant (*Borago officinalis* L.) or hawthorn (*Crataegus oxyacantha* L.) resulted in increased content of flavonoids and terpenoids in milk and, therefore, such polyphenol compounds entered into the milk may influence the quality and sensory traits of the milk [241]. The effect of including grape seed extract in Saanen goats’ diet was tested by Leparmarai et al. [242] who concluded that phenol concentrations in milk were increased by 32% after five weeks. This result notwithstanding, additional research may be advised to replicate this study and to use an alternate method for polyphenol determination in milk. The ability of goats to ingest higher phytochemical and polyphenol compounds are possible because of their coping mechanisms such as the higher salivary tannin-binding capacity [243], thus, providing them with the ability to survive on low-quality forage or rangeland vegetation.

Forages, particularly those given to goats, are generally rich in anti-nutritional factors, such as tannins. Such polyphenol compounds exert a wide array of effects on animal performance and health, from beneficial to milk production to toxic and ill, and even lethal effects [244]. The intake of condensed tannin was higher in Girgentana goats fed with fresh Sulla compared to hay with barley [237]. Feeding tannin-rich browse foliage species, i.e., lentisk, to Damascus dairy goats in confinement resulted in increasing milk protein, fat content, and curd firmness than compared to feeding vetch hay without lentisk foliage [245]. However, feeding on tannin-rich sources is known to reduce digestibility due to inhibiting rumen microbes, as shown by Woodward and Reed’s [246] comparison of two East African browse species, i.e., *Acacia brevispica* and *Sesbania sesban*, on feed digestibility. Goats that consumed *A. brevispica* showed lower digestibility of nitrogen due to higher tannin content than *S. sesban*. Additionally, the digestibility of fibre was lowered in a feed containing a higher content of *A. brevispica*, which is an important consideration for the palatability and rumen fill of goats. The inclusion of inert substances such as polyethylene glycol to goats’ feed can form a stable complex with tannins and, therefore, prevent the binding between tannins and proteins and thus minimise the negative effect of tannins. However, either the provision of polyethylene glycol to Sarda goats fed in metabolic cages or browsing in a Mediterranean scrubland did not influence the feed intake possibly due to higher allowances of metabolizable energy content. Nevertheless, milk production and milk urea content were increased with polyethylene glycol supplementation [247]. Similarly, providing polyethylene glycol to Sarda goats increased the of cis-9, trans-11 CLA, and trans-11 C18:1 in milk fat by 40% compared to the goats that were not provided with supplementation. This increase was further explained to be was associated with higher biohydrogenation activity of ruminal bacteria in goats supplemented with polyethylene glycol [248].

Goats’ αS1-casein polymorphism has received considerable attention due to its ability to modify protein and lipid composition of milk, which is of importance for its nutritional and technological properties. In Girgentana goats, homozygous and heterozygous individuals for the αS1-casein locus were tested for the inclusion of varying levels of fresh and dried forages by Giorgio et al. [249]. The authors reported an improved redox balance (i.e., lower oxidative stress) and albumin in goats fed with fresh Sulla forage compared to dried alfalfa forage, which indicate better animal health in Sulla-fed goats. Furthermore, genotype and diet showed interaction for glutathione peroxidase activity of goats’ blood samples, where glutathione peroxidase activity was higher in goats carrying strong αS1-casein homozygous alleles compared to those heterozygous for weak alleles. Studying the dietary energy level and αs1-casein polymorphism of Girgentana goats, Pagano et al. [250] showed that the intake of high energy content through feed resulted in higher milk yield and casein content in goats carrying stronger alleles (αS1-casein genotype) compared to weaker alleles. In Jamunapari goats, protein percentages in milk are significantly affected by αS1-casein polymorphism, with the highest in the AB genotype and lowest in the FF genotype [230].

Feed intake is particularly impacted by the quality of feed, as demonstrated by Silanikove [223] using high-quality roughage (e.g., alfalfa hay), medium quality roughage (e.g., Rhodes grass mixed with alfalfa), or low-quality roughage (e.g., wheat straw). The author showed an apparent dry matter digestibility of high-quality roughage (e.g., alfalfa hay) was higher in black Bedouin goats than Swiss Saanen goats when compared to low-quality roughage (e.g., wheat straw) [223]. This is because Bedouin goats have a greater digestive capacity and lower energy requirements that enable them to use, efficiently, high-fibre and low-nitrogen pastures from extremely arid areas. The lignification of plant cell walls is an important structural limitation factor that determines the carbohydrates’ digestibility in ruminants. The lignin content of grasses that are usually grazed by Spanish goats in Mexico showed a seasonal variation, where lignin content was lower in Spring and Autumn [251]. In fact, goats fed with low-quality roughage that are high in lignin undergo an extensive modification of ingested feed, such as a higher degree of degradation and absorption in the gastrointestinal tract, thus enhancing the availability of structural carbohydrates for microbial fermentation [223,252]. The literature suggests that the ability of goats to digest forages with elevated levels of phytochemicals provides a competitive advantage for ingesting a wide array of browse species that are generally rich in tannins and other phytochemicals and gives them an opportunity to survive in harsh environments with low availability of quality forages.

### 4.5. Buffalo—Milk Yield and Composition

The nutritional composition and properties of buffalo milk are known to vary with feeding systems, these, in turn, are influenced by on-farm factors and seasonal changes [253]. Buffalo can convert the carotene to vitamin A, resulting in whiter milk than cow milk. In traditional Indian buffalo farming, the predominant feed source is straw-based rations. This is because nutritionally balanced rations are not commonly available at the disposal of subsistence farmers in tropical regions (i.e., India, Sri Lanka, and Bangladesh). Frequently available feedstuffs include straw from various sources (e.g., wheat, paddy, gram, masoor, and soybean), uncultivated local grass, chari, maize fodder, cottonseed cake, and wheat bran, which is often fed in a combination of several materials [254]. Often, rations for buffalo in gestation are mostly straw with very little concentrates and fresh forages, without any mineral supplementation. Thus, strategic nutritional and mineral supplementation to buffalo prevent reproductive problems, metabolic disorders, and mastitis, yet do not affect the milk yield [255]. The replacement of cottonseed meal with coated (slow-releasing) urea is a beneficial strategy for improving the milk production of Murrah buffalo without altering milk protein or fat components [256] and thus providing economic, environmental, and production benefits to buffalo farmers. The incorporation of 10 kg of fresh sorghum into lactating buffalo’s diets demonstrated no effect on milk yield and composition, compared to buffalo fed the same diet without fresh sorghum. However, when the buffalo were provided with 20 kg of fresh sorghum (doubling the provision), more UFAs and less short-chain-SFAs were found in the milk [257].

A study by Tyagi et al. [258] compared the feeding of Berseem (*Trifolium alexandrinum*) fodder alone, concentrates alone, or a combination of concentrates and Berseem fodder to Murrah buffalo with ad libitum wheat straw. The authors reported no impact on milk yield or gross composition with the dietary inclusion of Berseem, except an increase of CLA content and the ratio of ω-3/ω-6 FAs were observed with the provision of Berseem fodder. Evaluating the protein status and nitrogen use efficiency in peri-urban milking buffalo in Pakistan, Habib et al. [259] reported that excess crude protein intake is associated with Berseem feeding and lower crude protein intake is associated with the feeding of maize fodder. Further, the provision of maize fodder resulted in higher milk yield, and improved milk urea-N and nitrogen use efficiency. Feeding *Moringa oleifera* leaf meal to buffalo, Tadeo et al. [260] reported no yield or compositional differences in buffalo milk derived from feeding *Moringa oleifera* or not. The supplementation of dietary antioxidants and micronutrients (Cu, Zn, and vitamins A and E) to Murrah buffalo resulted in lower probabilities of mastitis while improving the milk yield and milk fat and protein percentages [261]. The dietary supplementation of astaxanthin (carotenoid pigments) and prill fat (non-hydrogenated vegetable oil), in combination, increased the milk yield of buffalo while enhancing the physiological responses during heat stress [262]. This is an important consideration since buffalo are often exposed to heat stresses which could adversely impact production performance and milk yield. Seerapu et al. [263] demonstrated that the manipulation of microclimate (e.g., installation of foggers and air circulators) in the Murrah buffalo barns increased feed intake and thereby increased milk production, milk fat content, and solids-not-fat content as a result of decreased heat stress.

## 5. Conclusions and Future Opportunities

The diet of small and large ruminants can be managed to impact the quality of meat and milk products. This includes using nutritional interventions, such as differences in feeding strategies, production systems, concentrate feeding (feedlot), and type of supplementations (oils and meals) that, in turn, change the eating/consumption quality, nutritional value, and sensory attributes of meat and milk. The literature shows that animals reared under extensive production systems with pasture grazing deliver meat and milk with more nutritionally favourable traits (e.g., greater levels of ω-3 FAs and CLAs). In contrast, feeding concentrate-based diets high in hay, cereal grains, and by-products of oil industries (sunflower, safflower, and cotton meals) leads to more fat content and ω-6 FAs in meat and milk. The review indicates that the inclusion of oils rich in ω-3 FAs (e.g., canola oil, flaxseed oil, evening primrose oil, borage oil, algal oil, seal oil, or fish oil) can increase health-enhancing long-chain ω-3 PUFA profiles. However, with the increase in long-chain ω-3 PUFAs, the taste and aroma of meat and milk products may become compromised, unless antioxidants are optimised in the products for the avoidance of lipid oxidation, ensuring maximum storage quality. It should be noted that the extent of the effects of oils and oil meals supplementation on meat and milk flavour and oxidative stability depends on the duration of feeding, type of supplement, and concentration levels in the diets. Appropriate applications of antioxidant agents in feeds via on-farm and/or off-farm management are promising pathways to enhance shelf life, i.e., reduce the off-flavour development from lipid oxidation in meat (lamb, mutton, beef, or veal) and milk/milk products from sheep, goats, cattle, and buffalo. Balancing the dietary nutrients through the selection of available forages, filed crop residues, and agricultural by-products in a combination may be an option to elevate the quantity, quality, and nutritional value of meat and milk in a sustainable manner and maintaining a circular economy. The practicality and effectiveness of these strategies will drive future investigation and insight into the pathways for the nutritional enhancement of meat and milk products. In this pursuit, the fundamental action of any diet should not be forgotten. Specifically, all diets must support the growth and metabolic processes of ruminants by providing essential macro- and micronutrients matched to their stage of maturity (age), and production status or purposes.

## Figures and Tables

**Table 1:** Effect of different breeds (genotype) on carcass fatness (%) at four different slaughter ages ^1^. Each genotype grouping has 4 sires, with PDgrowth × Merino [PDg × M] and PDgrowth × (Border Leister × Merino) [PDg × (BL × M)] groupings sharing the same sires ^1^.

Age, Months	M × M	BL × M	PD × M		PDg × (BL × M)	SED		*p*-Values	
			PDg × M	PDm × M		PDg × M vs. PDg × (BL × M)	Other	Breed Combination	PDg × M vs. PDm × (BL × M)
4	15.2	17.7	16.4	16.6	19.4	0.86	1.01–1.09	0.001	0.86
8	19.7	22.8	21.7	21.1	25.0	0.55	1.01–1.07	0.001	0.55
14	22.9	29.7	26.9	26.0	29.6	0.68	1.31–1.36	0.001	0.51
22	29.1	37.1	32.4	32.3	36.2	0.75	1.08–1.17	0.001	0.92

^1^ Genotype details: M = Merino, BL = Border Leicester, PD = Poll Dorset, PDg = Poll Dorset sires selected for growth, PDm = Poll Dorset sires selected for muscling, BL × M = Border Leicester × Merino first cross, SED = standard error of the difference between means.

**Table 2:** Types and nutrient composition of feeds available to be fed to cattle ^1^.

Feed Source	DM	OM	CP	NDF	ADF
Alfalfa	85.5	n/a	15.0	54.2	36.8
Banana peel (sun dried)	87.8	n/a	8.3	47.9	29.8
Briquette from *Brachiaria*	90.9	n/a	5.3	73.3	50.7
Cactus pear	8.3	95.4	9.2	30.0	19.0
Cassava shoots hay	83.5	94.2	5.2	60.1	48.2
Citrus pulp	20.0	n/a	9.0	31.6	7.8
Corn ground grain	90.2	97.9	9.2	20.4	5.9
Crambe meal	91.7	93.4	36.3	28.0	16.7
*Cynodon nlemfuensis*	33.7	n/a	7.9	n/a	39.2
Elephant grass silage	51.3	n/a	6.6	71.2	42.7
Fresh sugarcane bagasse	71.0	96.7	1.9	83.4	57.9
Green maize	12.9	88.9	8.7	52.5	32.8
Lentisk	45.9	95.6	8.5	42.1	31.4
*Leucaena leucocephala*	30.3	n/a	25.9	n/a	24.8
Palm kernel cake	92.3	n/a	10.7	65.6	46.4
Peanut cake	90.9	n/a	44.7	11.8	7.0
*Pennisetum purpureum*	25.5	n/a	3.1	67.7	44.3
Rice polishing	92.0	n/a	8.1	17.8	32.2
Sorghum silage	33.2	92.2	6.6	65.0	38.9
Soybean meal	90.0	94.0	46.9	16.2	11.2
Sulla	19.1	n/a	17.2	44.3	26.9
*Tithonia diversifolia*	29.3	n/a	14.5	39.5	20.8
Wheat straw	90.1	91.5	3.1	77.8	50.4

^1^ n/a data not available, DM: dry matter, OM: organic matter, CP: crude protein, NDF: neutral detergent fibre, ADF: acid detergent fibre.

## Data Availability

Data are contained within the article.
